# Supplementary material for: Predictors of unwillingness to receive COVID -19 vaccines among Ethiopian Medical students
Source: PLoS One. 2022 Nov 2;17(11):e0276857. doi: 10.1371/journal.pone.0276857 (PMC9629586; doi:10.1371/journal.pone.0276857)
Supplement: S1 Questionnaire — (DOCX) [file pone.0276857.s001.docx]

| Questionnaire **CONSCENT FORM**  My name is………………We are collecting data on **Unwillingness to receive COVID -19 vaccines among Ethiopian Medical student** for a research purpose. This form explains why we are going to do this study, your role in the study, the benefits and risks of involving in this study, compensations and confidentiality of the information you give us.  **Purpose-** This study is assessing Unwillingness to receive COVID -19 vaccines among Ethiopian Medical student  **Procedures** - The researcher will conduct an interview and the interview will last about 30-35 minutes. The interview will take place anywhere in the compound, which is comfortable for the interview process and for the participant. You will be asked questions about socio-demographic issues, knowledge, attitude about COVID -19 vaccines  **Risks associated with the study**- Apart from the time you spend with us there we will not be any risk that you are exposed by participating in this study. If there is any question you don’t want to answer, you can miss out that question.  **Benefits of the study-** We expect that the study will help to withdraw attention of concerned bodies in order to emphasize more on strategies and interventions which address issue of medical student need regarding COVID -19 vaccines.  **Compensations-** There will not be any monetary incentive for your time.  **Confidentiality of your information-** The information you give during this study will be held confidential. Once the data is entered into a computer, it will be coded and becomes anonymous. Your personal information will never be disclosed in either oral or written form.  **Termination of the study-** You will only be selected based on your willingness and without any obligation to participate in the study. You have also the full right to withdraw your participation at any time before completing the interview.  I would also like to inform you that this study is approved by the ethical committees of Mizan Tepi University college of health science.  **If you agree to participate put your sign here** | | | | | | |
| --- | --- | --- | --- | --- | --- | --- |
| **Items used to measure demographic background, health status and COVID-19 experience** | | | | | | |
|  | What is your Gender? | 1. Male 2. Female | | | | |
|  | What is your Age? |  | | | | |
|  | What is your Religion? | 1. Orthodox 2. Protestant 3. Muslim | | | | |
|  | Place of origin | 1. Urban 2. Rural | | | | |
|  | What is your Department? |  | | | | |
|  | How long did you stay in university | 1^st^ year 2^nd^ Year 3^rd^ Year 4^th^ Year 5^th^ year | | | | |
|  | What is your Cumulative CGPA |  | | | | |
|  | Do you have any Chronic illness | 1. Yes 2. No | | | | |
|  | How do you rate your health | 1 very poor 2. Poor 3. Okay 4. good 5.verry good | | | | |
| **Items used to measure Knowledge, risk factor, and preventive behavior of COVID-19** | | | | | | |
| **I. Knowledge about transmission(which one of the following is the transmission rout of COVID 19)** | | | | Yes | | No |
|  | Inhalation | | |  | |  |
|  | Large droplet | | |  | |  |
|  | Kissing | | |  | |  |
|  | handshaking | | |  | |  |
|  | Touching contaminated item | | |  | |  |
|  | From air | | |  | |  |
|  | Skin contact | | |  | |  |
|  | Fecal or oral rout | | |  | |  |
|  | Animals | | |  | |  |
|  | Contaminated food | | |  | |  |
| **II. Knowledge about risk factors (which one of the following is the Risk factors for COVID 19)** | | | |  | |  |
|  | People with chronic disease are at high risk of COVID-19 | | |  | |  |
|  | Only sick people should wear mask to prevent the spread of the disease | | |  | |  |
|  | Wearing mask regularly prevents getting the disease | | |  | |  |
|  | I believe that vaccine can prevent the spread of COVID | | |  | |  |
|  | COVID-19 has high recovery rate with 90%recovery | | |  | |  |
|  | One sick people can transmit the disease to four other people | | |  | |  |
|  | pregnant women’s are at high risk of COVID 16 | | |  | |  |
|  | Children are at high risk of COVID 16 | | |  | |  |
| **III. Knowledge about preventive behavior((which one of the following is prevention behavior of COVID 19))** | | | | | | |
|  | Covering face when coughing and sneezing | | |  | |  |
|  | Avoiding crowed areas | | |  | |  |
|  | Wearing face mask | | |  | |  |
|  | Avoiding close contact | | |  | |  |
|  | Avoiding large gathering | | |  | |  |
|  | Avoid touching face | | |  | |  |
|  | Using disinfectants | | |  | |  |
|  | Staying home | | |  | |  |
|  | Washing hand for 20-60 second with soap | | |  | |  |
| Knowledge about COVID 19 vaccine | | | | | | |
|  | Do you know where COVID 19 vaccine is given in Ethiopia | | |  | |  |
|  | Do you know you cannot receive COVID 19 vaccine from pharmacy | | |  | |  |
|  | Do you know you can receive COVID 19 vaccine | | |  | |  |
|  | From where you heard about COVID 19 vaccine | | |  | |  |
|  | Side effect of COVID 19 vaccine do not last more than 5 days | | |  | |  |
|  | COVID 19 vaccine have mild side effect | | |  | |  |
|  | COVID 19 vaccine safe for children | | |  | |  |
|  | COVID 19 vaccine is safe for pregnant women | | |  | |  |
|  | Do you know how may COVID 19 vaccine dose you should take | | |  | |  |
|  | Do you know how long COVID 19 vaccine can be effective | | |  | |  |
| Opinion about COVID 19 vaccine | | | |  | |  |
|  | I think COVID 19 vaccine probably will not work | | |  | |  |
|  | I do not trust COVID 19 vaccine | | |  | |  |
|  | I think COVID 19 vaccine not necessary | | |  | |  |
|  | I think it is not important to get vaccine | | |  | |  |
|  | | | | | | |
|  | | | | | | |
|  | | | | | | |
| **Items used to measure HBM, TPB, and 5C psychological antecedents.** | | | | | | |
| **THE HEALTH BELIEF MODEL** | | *Strongly*  *disagree* | *disagree* | *Neutral* | *agree* | *Strongly agree* |
| **Perceived susceptibility** | |  |  | | | |
|  | I am worried about the likelihood of getting infected by COVID-19 |  |  |  |  |  |
|  | I am at high risk of COVID-19 because of my health conditions |  |  |  |  |  |
| **Perceived severity** | |  |  |  |  |  |
|  | I will be very sick if I get infected by COVID-19 |  |  |  |  |  |
|  | I am very concerned that I could die from COVID-19 |  |  |  |  |  |
| **Perceived benefits** | |  |  |  |  |  |
|  | I think vaccination is good because it will make me less worried about COVID-19 |  |  |  |  |  |
|  | I believe vaccination will decrease my risk of getting infected by COVID-19 |  |  |  |  |  |
|  | I think the complications of COVID-19 will decrease if I get vaccinated and then get infected with the Coronavirus. |  |  |  |  |  |
| **Perceived barriers** | |  |  |  |  |  |
|  | I am worried that the possible side effects of the COVID-19 vaccination would interfere with my usual activities |  |  |  |  |  |
|  | I am concerned about the efficacy of the COVID-19 vaccine |  |  |  |  |  |
|  | I have a concern that I may receive faulty/fake COVID-19 vaccine |  |  |  |  |  |
|  | It concerns me that the development of a COVID-19 vaccine is too rushed to test its safety properly |  |  |  |  |  |
|  | I am concerned about the long-term side effects of the COVID-19 vaccination |  |  |  |  |  |
| **Cues to action** | |  |  | |  | |
|  | I have been infected with COVID-19 |  |  |  |  |  |
|  | I have a relative who hospitalized or died from COVID-19 |  |  |  |  |  |
|  | I had been caring for someone with COVID-19 Infection |  |  |  |  |  |
|  | Where have you heared about COVID 19 vaccine | facebook | Gov;t media | News peper | Health care worker | Family/freind |
| **willingness to receive COVID 19 vaccine** | | | | | | |
|  | I intend to receive vaccine against COVID-19 |  |  |  |  |  |
|  | I predict I will receive vaccine against COVID-19 |  |  |  |  |  |
|  | I plan to receive vaccine against COVID-19 |  |  |  |  |  |
| ***If you are intended to be vaccinated what motives you to be vaccinated?(TICK your answer)*** | | | | | | |
|  | I am at risk of COVID-19 infection. | | | |  |  |
|  | I don’t want to be infected. | | | |  |  |
|  | I don’t want to transmit COVID-19 to others. | | | |  |  |
|  | I want to be an actor in the fight against COVID-19. | | | |  |  |
|  | I trust in the efficacy of the COVID-9 vaccine | | | |  |  |
| ***If you are not intended to be vaccinated what is your reasons of refusal?*** | | | | | | |
|  | I am not at risk of sever COVID-19 infection. | | | |  |  |
|  | I am not really scared of being infected by COVID-19. | | | |  |  |
|  | I prefer to wait until I have more experience with these new vaccines. | | | |  |  |
|  | I doubt the efficacy of the vaccine. | | | |  |  |
|  | I am afraid of mild side effects (e*.g., fever, pain at the injection site) of the vaccine*. | | | |  |  |
|  | I fear serious side effects (*e.g., hospitalization, serious illness*) of the vaccine. | | | |  |  |
