# Supplementary material for: Predictors of unwillingness to receive COVID -19 vaccines among Ethiopian Medical students
Source: PLoS One. 2022 Nov 2;17(11):e0276857. doi: 10.1371/journal.pone.0276857 (PMC9629586; doi:10.1371/journal.pone.0276857)
Supplement: Attachment — Submitted filename: Review comments.docx [file pone.0276857.s003.docx]

**Summary:** The study topic is relevant. The design is overall good but needs some clarifications in certain sections.

**Overall impression:** Some doubts have to be cleared.

**Major issues:**

The semantic error and lack of abbreviations, mostly in the result and discussion sections have made the manuscript difficult to comprehend.

Method section, subsection “Sample size determination, and sampling technique”: Please mention the formula.

Is the questionnaire validated and pre-tested?

**Minor issues:**

Result section, 349 line number: Is it percentage and number in the bracket?

Please elaborate the discussion by adding some contrasting findings to that of other studies.
