# Supplementary material for: Predictors of unwillingness to receive COVID -19 vaccines among Ethiopian Medical students
Source: PLoS One. 2022 Nov 2;17(11):e0276857. doi: 10.1371/journal.pone.0276857 (PMC9629586; doi:10.1371/journal.pone.0276857)
Supplement: Attachment — Submitted filename: Response to Reviewers.docx [file pone.0276857.s004.docx]

# Journal requirements:

1. Please ensure that your manuscript meets PLOS ONE's style requirements, including those for file naming. The PLOS ONE style templates can be found at

<https://journals.plos.org/plosone/s/file?id=wjVg/PLOSOne_formatting_sample_main_body.pdf> and <https://journals.plos.org/plosone/s/file?id=ba62/PLOSOne_formatting_sample_title_authors_affiliations.pdf>.

**Response: Editors of journals, We've acknowledged the suggestion and updated the manuscript to comply with PLOS ONE's style guidelines. That is the Author, affiliation formatting guide line, which is used to change the title page, and the manuscript body formatting guide line, which is used to change the manuscript body. While doing so, the above-mentioned URL came in handy, and we'd like to thank the journal team for providing these rules.**

2. We suggest you thoroughly copyedit your manuscript for language usage, spelling, and grammar. If you do not know anyone who can help you do this, you may wish to consider employing a professional scientific editing service.

Whilst you may use any professional scientific editing service of your choice, PLOS has partnered with both American Journal Experts (AJE) and Editage to provide discounted services to PLOS authors. Both organizations have experience helping authors meet PLOS guidelines and can provide language editing, translation, manuscript formatting, and figure formatting to ensure your manuscript meets our submission guidelines. To take advantage of our partnership with AJE, visit the AJE website (<http://learn.aje.com/plos/>) for a 15% discount off AJE services. To take advantage of our partnership with Editage, visit the Editage website ([www.editage.com](http://www.editage.com/)) and enter referral code PLOSEDIT for a 15% discount off Editage services.  If the PLOS editorial team finds any language issues in text that either AJE or Editage has edited, the service provider will re-edit the text for free.

**Response: We have completely copyedited the manuscript for language use, spelling, and grammar, and we appreciate you for your patience.**

Upon resubmission, please provide the following:

The name of the colleague or the details of the professional service that edited your manuscript

A copy of your manuscript showing your changes by either highlighting them or using track changes (uploaded as a *supporting information* file)

A clean copy of the edited manuscript (uploaded as the new *manuscript* file

3. Please provide additional details regarding participant consent.

In the ethics statement in the Methods and online submission information, please ensure that you have specified (1) whether consent was informed and (2) what type you obtained (for instance, written or verbal, and if verbal, how it was documented and witnessed). If your study included minors, state whether you obtained consent from parents or guardians. If the need for consent was waived by the ethics committee, please include this information.

If you are reporting a retrospective study of medical records or archived samples, please ensure that you have discussed whether all data were fully anonymized before you accessed them and/or whether the IRB or ethics committee waived the requirement for informed consent. If patients provided informed written consent to have data from their medical records used in research, please include this information

**Response: Thank you, editors, for your advice with the ethical statement. We utilized written informed consent. As a result, informed written consent was acquired directly from participants, all of whom were above the age of 18. we have included in the manuscript as follow “**This study was conducted in harmony with the Declaration of Helsinki. Mizan Tepi University Research Ethics Review Committee approved it. All participant were assured the autonomy to participate or not, the a written informed consent was sought from each participant.”

4. Thank you for stating the following financial disclosure:

“no fund”

At this time, please address the following queries:

a) Please clarify the sources of funding (financial or material support) for your study. List the grants or organizations that supported your study, including funding received from your institution.

b) State what role the funders took in the study. If the funders had no role in your study, please state: “The funders had no role in study design, data collection and analysis, decision to publish, or preparation of the manuscript.”

c) If any authors received a salary from any of your funders, please state which authors and which funders.

d) If you did not receive any funding for this study, please state: “The authors received no specific funding for this work.

Please include your amended statements within your cover letter; we will change the online submission form on your behalf.

**Response: The authors received no specific funding for this work**

5. Please amend your authorship list in your manuscript file to include author Eyob Bekele.

**Response: Thank you, dear editors, for recommending that the authorship list be updated by adding author Eyob Bekele to the text file.**

6. Please include your full ethics statement in the ‘Methods’ section of your manuscript file. In your statement, please include the full name of the IRB or ethics committee who approved or waived your study, as well as whether or not you obtained informed written or verbal consent. If consent was waived for your study, please include this information in your statement as well.

**Response: thank you dear editors, we have included the ethical statement in the method section, as follow “**This study was conducted in harmony with the Declaration of Helsinki. Mizan Tepi University Research Ethics Review Committee approved it. All participant were assured the autonomy to participate or not, the a written informed consent was sought from each participant.”

7. Please include captions for your Supporting Information files at the end of your manuscript, and update any in-text citations to match accordingly. Please see our Supporting Information guidelines for more information: <http://journals.plos.org/plosone/s/supporting-information>.

**Response: Thank you, dear editor; we've added captions to the Supporting Information files at the end of the paper, and we've updated any in-text citations to match.**

Additional Editor Comments:

I request authors to revise the manuscript based on reviewers' comments. In addition, the Ethical approval details should be mentioned in the manuscript. The abstract should be formatted as per the journal style. The questionnaire validation should be discussed in detail. The discussion section appears weak and should be rewritten to make stronger arguments and conclusions.
[Note: HTML markup is below. Please do not edit.]

**Response: Thank you very much, editor. The majority of the comments from reviewers and editors were accepted. We've also made the necessary changes to the document. The ethical approval statement is included in the manuscript, the abstract part is organized according to journal style, and the validity of the questionnaire was also explored in detail. Finally, we attempted to modify the section on discussion.**

# Reviewers' comments: Reviewer's Responses to Questions

**Comments to the Author**

1. Is the manuscript technically sound, and do the data support the conclusions?

The manuscript must describe a technically sound piece of scientific research with data that supports the conclusions. Experiments must have been conducted rigorously, with appropriate controls, replication, and sample sizes. The conclusions must be drawn appropriately based on the data presented.

Reviewer #1: Yes

Reviewer #2: Yes

2. Has the statistical analysis been performed appropriately and rigorously?

Reviewer #1: Yes

Reviewer #2: Yes

3. Have the authors made all data underlying the findings in their manuscript fully available?

The [PLOS Data policy](http://www.plosone.org/static/policies.action#sharing) requires authors to make all data underlying the findings described in their manuscript fully available without restriction, with rare exception (please refer to the Data Availability Statement in the manuscript PDF file). The data should be provided as part of the manuscript or its supporting information, or deposited to a public repository. For example, in addition to summary statistics, the data points behind means, medians and variance measures should be available. If there are restrictions on publicly sharing data—e.g. participant privacy or use of data from a third party—those must be specified.

Reviewer #1: Yes

Reviewer #2: No

**Response: Dear Reviewer, thank you for your time and consideration. the data or supporting material supplied as part of the paper**

4. Is the manuscript presented in an intelligible fashion and written in standard English?
PLOS ONE does not copyedit accepted manuscripts, so the language in submitted articles must be clear, correct, and unambiguous. Any typographical or grammatical errors should be corrected at revision, so please note any specific errors here.

 Reviewer #1: No

Reviewer #2: No

**Response: Dear Reviewer, we have updated the English language to meet normal English requirements.**

5. Review Comments to the Author

Please use the space provided to explain your answers to the questions above. You may also include additional comments for the author, including concerns about dual publication, research ethics, or publication ethics. (Please upload your review as an attachment if it exceeds 20,000 characters)

Reviewer #1: The statistical method used is correct. Adequate analyses have been performed. The manuscript is unintelligible in some places. The authors have made all data underlying the findings in their manuscript available if needed.

**Response: Regards, Reviewer We've modified the English language to suit Standard English criteria, making it more understandable.**

Reviewer #2: The title is Ok.
ABSTRACTS
There are so many typographical errors . Example: "In this study 145(40%) of medical student were not willing to receive COVID-19 vaccination. And also age of student .43(.29,.63), Knowledge status about COVID 19 infection transmission, risk factors and control behavior 1.45(1.14, 1.85), perceived susceptibility, perceived severity perceived benefit, positive attitude and confidence on safety and efficacy of the vaccine were predictors of non-willingness to receive COVID 19 vaccine."
How can age be .43?
There are so many unnecessary use of upper case. Example: 'Therefore, this study assessed Predictors of Unwillingness to receive COVID -19 vaccines among Ethiopian Medical student. "

**Response:** thank you dear reviewer we would like to apologize for the typographical errors, we have tried to correct it. We have also corrected unnecessary use of upper cases.
INTRODUCTION
the authors should beef up the justification for the study

**Response:** we have tried to add more justification for the study
RESULTS
The authors should include a flow chart showing the participants flow in the study.

**Response** :

6. PLOS authors have the option to publish the peer review history of their article ([what does this mean?](https://journals.plos.org/plosone/s/editorial-and-peer-review-process#loc-peer-review-history)). If published, this will include your full peer review and any attached files.


If you choose “no”, your identity will remain anonymous but your review may still be made public.


**Do you want your identity to be public for this peer review?** For information about this choice, including consent withdrawal, please see our [Privacy Policy](https://www.plos.org/privacy-policy).

Reviewer #1: No

Reviewer #2: No

[NOTE: If reviewer comments were submitted as an attachment file, they will be attached to this email and accessible via the submission site. Please log into your account, locate the manuscript record, and check for the action link "View Attachments". If this link does not appear, there are no attachment files.]

While revising your submission, please upload your figure files to the Preflight Analysis and Conversion Engine (PACE) digital diagnostic tool, <https://pacev2.apexcovantage.com/>. PACE helps ensure that figures meet PLOS requirements. To use PACE, you must first register as a user. Registration is free. Then, login and navigate to the UPLOAD tab, where you will find detailed instructions on how to use the tool. If you encounter any issues or have any questions when using PACE, please email PLOS at [figures@plos.org](mailto:figures@plos.org). Please note that Supporting Information files do not need this step.
