# Supplementary material for: Predictors of unwillingness to receive COVID -19 vaccines among Ethiopian Medical students
Source: PLoS One. 2022 Nov 2;17(11):e0276857. doi: 10.1371/journal.pone.0276857 (PMC9629586; doi:10.1371/journal.pone.0276857)
Supplement: Attachment — Submitted filename: Response to Reviewers.docx [file pone.0276857.s005.docx]

**A rebuttal letter that responds to each point raised by the academic editor and reviewer(s)**

PONE-D-21-36100R1

Predictors of Unwillingness to receive COVID -19 vaccines among Ethiopian Medical student

**Editors comment**

PLOS ONE

Dear Dr. Getachew,

Thank you for submitting your manuscript to PLOS ONE. After careful consideration, we feel that it has merit but does not fully meet PLOS ONE’s publication criteria as it currently stands. Therefore, we invite you to submit a revised version of the manuscript that addresses the points raised during the review process.

Please submit your revised manuscript by Sep 24 2022 11:59PM. If you will need more time than this to complete your revisions, please reply to this message or contact the journal office at [plosone@plos.org](mailto:plosone@plos.org). When you're ready to submit your revision, log on to <https://www.editorialmanager.com/pone/> and select the 'Submissions Needing Revision' folder to locate your manuscript file.

Please include the following items when submitting your revised manuscript:

- A rebuttal letter that responds to each point raised by the academic editor and reviewer(s). You should upload this letter as a separate file labeled 'Response to Reviewers'.
- A marked-up copy of your manuscript that highlights changes made to the original version. You should upload this as a separate file labeled 'Revised Manuscript with Track Changes'.
- An unmarked version of your revised paper without tracked changes. You should upload this as a separate file labeled 'Manuscript'.

**Author response:** Hello reviewer I appreciate the advice and criticism you provided. When the first academic editor left, my colleague and I were concerned. However, now that you are here and have provided such insightful scientific feedback, we are pleased to have you and your help enhance this work as a whole. Additionally, we are ready to consider all suggestions or comments.

**Editors comment**

If you would like to make changes to your financial disclosure, please include your updated statement in your cover letter.

**Author response: Regarding financial disclosure, as the amount is about equal to our yearly gross income, we have requested publication support.**

**Editors comment**

Guidelines for resubmitting your figure files are available below the reviewer comments at the end of this letter.

**Author response: Thank you, Editor; we followed the instructions to submit figure files; we found the PACE to be extremely fascinating; we appreciate your advice.**

**Editors comment**

If applicable, we recommend that you deposit your laboratory protocols in [protocols.io](http://protocols.io/) to enhance the reproducibility of your results. Protocols.io assigns your protocol its own identifier (DOI) so that it can be cited independently in the future. For instructions see: <https://journals.plos.org/plosone/s/submission-guidelines#loc-laboratory-protocols>. Additionally, PLOS ONE offers an option for publishing peer-reviewed Lab Protocol articles, which describe protocols hosted on [protocols.io](http://protocols.io/). Read more information on sharing protocols at <https://plos.org/protocols?utm_medium=editorial-email&utm_source=authorletters&utm_campaign=protocols>.

**Author response: Thank you, Editor; but I don’t think this is applicable for this manuscript.**

We look forward to receiving your revised manuscript.

Kind regards,

Dragan Pamucar
Academic Editor
PLOS ONE

**Journal Requirements:**

Please review your reference list to ensure that it is complete and correct. If you have cited papers that have been retracted, please include the rationale for doing so in the manuscript text, or remove these references and replace them with relevant current references. Any changes to the reference list should be mentioned in the rebuttal letter that accompanies your revised manuscript. If you need to cite a retracted article, indicate the article’s retracted status in the References list and also include a citation and full reference for the retraction notice.

[Note: HTML markup is below. Please do not edit.]

**Author response:** My dear reviewer, While searching through our reference list, we were unable to locate a retracted article. Inappropriately mentioned articles have been amended and removed, too.

Reviewers' comments:

Reviewer's Responses to Questions

**Comments to the Author**

1. If the authors have adequately addressed your comments raised in a previous round of review and you feel that this manuscript is now acceptable for publication, you may indicate that here to bypass the “Comments to the Author” section, enter your conflict of interest statement in the “Confidential to Editor” section, and submit your "Accept" recommendation.

Reviewer #1: (No Response)

Reviewer #2: (No Response)

2. Is the manuscript technically sound, and do the data support the conclusions?

The manuscript must describe a technically sound piece of scientific research with data that supports the conclusions. Experiments must have been conducted rigorously, with appropriate controls, replication, and sample sizes. The conclusions must be drawn appropriately based on the data presented.

Reviewer #1: Partly

Reviewer #2: Yes

3. Has the statistical analysis been performed appropriately and rigorously?

Reviewer #1: Yes

Reviewer #2: Yes

4. Have the authors made all data underlying the findings in their manuscript fully available?

The [PLOS Data policy](http://www.plosone.org/static/policies.action#sharing) requires authors to make all data underlying the findings described in their manuscript fully available without restriction, with rare exception (please refer to the Data Availability Statement in the manuscript PDF file). The data should be provided as part of the manuscript or its supporting information, or deposited to a public repository. For example, in addition to summary statistics, the data points behind means, medians and variance measures should be available. If there are restrictions on publicly sharing data—e.g. participant privacy or use of data from a third party—those must be specified.

Reviewer #1: (No Response)

Reviewer #2: Yes

5. Is the manuscript presented in an intelligible fashion and written in standard English?

PLOS ONE does not copyedit accepted manuscripts, so the language in submitted articles must be clear, correct, and unambiguous. Any typographical or grammatical errors should be corrected at revision, so please note any specific errors here.

Reviewer #1: No

Reviewer #2: Yes

6. Review Comments to the Author

Please use the space provided to explain your answers to the questions above. You may also include additional comments for the author, including concerns about dual publication, research ethics, or publication ethics. (Please upload your review as an attachment if it exceeds 20,000 characters)

Reviewer #1: a. The grammatical errors and English language correction is to be done.

**Author response: Thank you, reviewer. We have removed any grammatical errors, corrected them, and copy edited the English language**
b. The discussion still seems incomplete.

**Author response: Reviewer, I appreciate your time. The discussion section has been widened and elaborated where necessary.**

Reviewer #2: The authors should remove the ampersand in 'Study area & period"
The authors should endeavor to add flow chart showing the participants flow.

**Author response: Please accept our apologies for putting the ampersand in the incorrect place. Thank you. Regarding putting the flow chart we added the flow chart.**

7. PLOS authors have the option to publish the peer review history of their article ([what does this mean?](https://journals.plos.org/plosone/s/editorial-and-peer-review-process#loc-peer-review-history)). If published, this will include your full peer review and any attached files.


If you choose “no”, your identity will remain anonymous but your review may still be made public.
**Author response:**

**Do you want your identity to be public for this peer review?** For information about this choice, including consent withdrawal, please see our [Privacy Policy](https://www.plos.org/privacy-policy).

Reviewer #1: No

Reviewer #2: **Yes:** George Eleje

[NOTE: If reviewer comments were submitted as an attachment file, they will be attached to this email and accessible via the submission site. Please log into your account, locate the manuscript record, and check for the action link "View Attachments". If this link does not appear, there are no attachment files.]

While revising your submission, please upload your figure files to the Preflight Analysis and Conversion Engine (PACE) digital diagnostic tool, <https://pacev2.apexcovantage.com/>. PACE helps ensure that figures meet PLOS requirements. To use PACE, you must first register as a user. Registration is free. Then, login and navigate to the UPLOAD tab, where you will find detailed instructions on how to use the tool. If you encounter any issues or have any questions when using PACE, please email PLOS at [figures@plos.org](mailto:figures@plos.org). Please note that Supporting Information files do not need this step.

**Author response: Thank you, Editor; we followed the instructions to submit figure files; we found the PACE to be extremely fascinating; we appreciate your advice.**

*In compliance with data protection regulations, you may request that we remove your personal registration details at any time.* [*(Remove my information/details)*](https://www.editorialmanager.com/pone/login.asp?a=r)*. Please contact the publication office if you have any questions.*

|  | ReplyForward |
| --- | --- |

**Reviewer comment from the action link "View Attachments"**

**Summary:** The study topic is relevant. The design is overall good but needs some clarifications in certain sections.

**Overall impression:** Some doubts have to be cleared.

**Author Response:** Thank you dear reviewer, you have given us constructive comment we have included your comment in the revised manuscript to clarify certain section.

**Major issues:**

**Reviewer comment:** The semantic error and lack of abbreviations, mostly in the result and discussion sections have made the manuscript difficult to comprehend.

**Author Response:** Thank you dear reviewer, we apologize for not included list of abbreviation, now in the revised version of the manuscript list of abbreviation was included.

**Reviewer comment:** Method section, subsection “Sample Size determination and sampling technique”: Please mention the formula.

**Author Response:** Thank you dear reviewer, we have mention the formula as single population proportion formula and we have included the formula.

**Reviewer comment :**Is the questionnaire validated and pre-tested?

**Author Response:** yes the questionnaire is pretested and validated

**Minor issues:**

**Reviewer comment**: Result section, 349 line number: Is it percentage and number in the bracket?

**Author Response:** Thank you dear reviewer, comment accepted the number in the bracket it is percentage and corrected accordingly 207(67.9%).

**Reviewer comment**: Please elaborate the discussion by adding some contrasting findings to that of other studies.

**Author Response: Thank you dear reviewer, we have accepted your comment and elaborated the discussion by adding some contrasting finding.**
